# Molecular analysis of HBV pre-core gene mutations in patients co-infected with HIV at a tertiary care hospital in North India

**DOI:** 10.1099/acmi.0.000927.v4

**Published:** 2025-08-05

**Authors:** Hiba Sami, Mohd Asaad, Safiya Firoze, Syed Haider Mehdi Husaini, Parvez A. Khan, Nazish Fatima, Adil Raza, Haris M. Khan

**Affiliations:** 1 Department of Microbiology, Jawaharlal Nehru Medical College and Hospital (JNMCH), Aligarh Muslim University (AMU), Aligarh, India; 2 Department of Medicine, Jawaharlal Nehru Medical College and Hospital (JNMCH), Aligarh Muslim University (AMU), Aligarh, India

**Keywords:** hepatitis B virus, hepatitis B virus and human immunodeficiency virus (HBV-HIV) co-infection, pre-core (PC) mutation

## Abstract

**Objective.** Hepatitis B virus (HBV) spontaneous mutations may impact the severity of liver disease. This study aimed to assess the mutations in the pre-core (PC) region in HBV-HIV (human immunodeficiency virus) co-infected patients. Additionally, we explored its association with genotypes and examined the clinical implications.

**Methods.** A total of 100 HBV-HIV co-infected patients and 50 HBV mono-infected patients were included in the study. We focused on the PC region of the HBV genome, sequencing it to identify PC mutant variants. PCR products were quantified via spectrophotometry and sequenced using the Sanger method. The resulting sequences were assembled, annotated and aligned in a single reading frame. Subsequent mutational and phylogenetic analyses were performed using UGENE software to determine the genotypes of the isolates.

**Results.** The PC region was successfully amplified and sequenced in 27 samples, comprising 16 from HBV-HIV co-infected patients and 11 from HBV mono-infected patients. Phylogenetic analysis identified two HBV genotypes: genotype D, which was predominant and found in 24 samples (88.9%), and genotype A, present in 3 samples (11.1%). A T-to-C mutation at nucleotide position 1912 was detected in 48.1% of the patients. Furthermore, several additional PC mutations were observed, including A1850T, C1858T, G1899A, G1862T, G1951T, T1812C and T1809G, along with novel mutations such as C1936T, A2011G, T2020A and C2044T. Notably, the prevalence of these PC mutations did not significantly differ between the HBV mono-infected and HBV-HIV co-infected groups.

**Conclusion.** This study underscored the prevalence of PC mutations in HBV-HIV co-infected patients. Although several of these mutations have been previously reported, our findings also revealed novel variants. Further research is needed to elucidate the clinical significance of these new mutations.

## Data Summary

The sequence data in this study were generated by Sanger sequencing, and the sequence data have been archived and are freely available on GenBank under the accession numbers PP690941 to PP690967.

## Introduction

Despite the availability of an effective vaccination, hepatitis B virus (HBV) infection continues to be an international health issue, with 400 million individuals globally suffering from it chronically [1]. This virus is composed of partially dsDNA and belongs to the *Hepadnaviridae* family. It has four ORF regions that overlap, encoding four genes: C, P, S and X. Gene C codes for the core protein. The pre-core (PC) protein is created from an upstream in-frame AUG start codon that comes before the start codon of the C gene (HBcAg). The PC protein is processed by proteases to form HBV e-antigen (HBeAg). Gene P codes for the enzyme DNA polymerase. HBV surface antigen (HBsAg), the surface antigen, is encoded by the gene S. Although the HBsAg gene is a single long ORF, it is divided into pre-S1, pre-S2 and S by three in-frame ‘start’ (ATG) codons. The presence of these start codons results in the production of three distinctly sized polypeptides: small (S), middle (pre-S2+S), and large (pre-S1+pre-S2+S) [2].

Adjacent to the 5′ end of the viral genome’s core ORF, there is a concise, in-phase ORF responsible for a larger protein than the core, known as the PC protein. It undergoes cleavage at the 5′ end via signal peptidase and additional processing at the 3′ tail, leading to the formation of HBeAg. HBeAg is an indicator for active HBV proliferation; emergence of anti-HBe can signify replication cessation in the liver, implying lower infectivity [3].

HBV is categorized into eight genotypes, labelled A to H. These are based on intergroup divergence of ≥8% in the entire nt sequence [4]. The HBV genotype affects the prevalence of PC variation; genotype D has the highest frequency of this variant, while genotype A has the lowest [5]. There is currently much interest in the relationship between HBV genotypes, the severity of the disease, and the response to treatment. Most data available come from Eastern Asian countries, with a general predominance of genotypes B and C [6,7]. Variant viruses significantly impact the natural history of the clinical disease. In regions like the Mediterranean and the Far East, infection with HBV variants that fail to synthesize HBeAg is common among those with chronic liver disease (CLD) [8]. The prevalence of PC region mutations, along with their correlation with HBV genotypes and disease severity, remains unclear in the context of Indian patients [9,10]. According to reports, mutant HBV viruses are responsible for 25% of CLD cases involving hepatitis B among Asian Indians. Surface variants account for 10% of these viruses and PC variants for up to 15% of cases [10,11]. Mediterranean nations have a high frequency of HBeAg-negative chronic hepatitis B (CHB), and several subpopulations have been found to have altered PC regions, which prevent the synthesis of HBeAg [12,13]. However, PC mutations have also been identified in HBeAg-positive individuals, suggesting the coexistence of both mutant and WT HBV strains within the same host [14].

Given their similar methods of transmission, co-infections with HBV are not uncommon among those infected with human immunodeficiency virus (HIV) [15]. For instance, advanced liver conditions such as chronic cirrhosis and hepatocellular carcinoma (HCC) in HBV mono-infection have been associated with the well-characterized A1762T/G1764A double mutation in the basal core promoter (BCP) region and the G1896A mutation in the PC region. These mutations occur within the BCP (nt positions 1742–1849) and PC (positions 1814–1900) regions of the HBV genome, respectively, and are commonly referenced relative to the EcoRI restriction site for consistency in HBV molecular mapping [16,17]. Pre-S1/S2 deletion has likewise shown linkage to higher HCC risk in cases of mono-HBV infection in addition to advancing liver disease. Furthermore, studies have shown that people who have both HIV and HBV are far more inclined to carry pre-S2 deletions than people who only have HBV [18,19].

This study aimed to assess the mutations in the PC region. Additionally, we explored its association with genotypes and examined the clinical implications related to patients co-infected with HBV and HIV.

## Methods

**Fig. 1.**
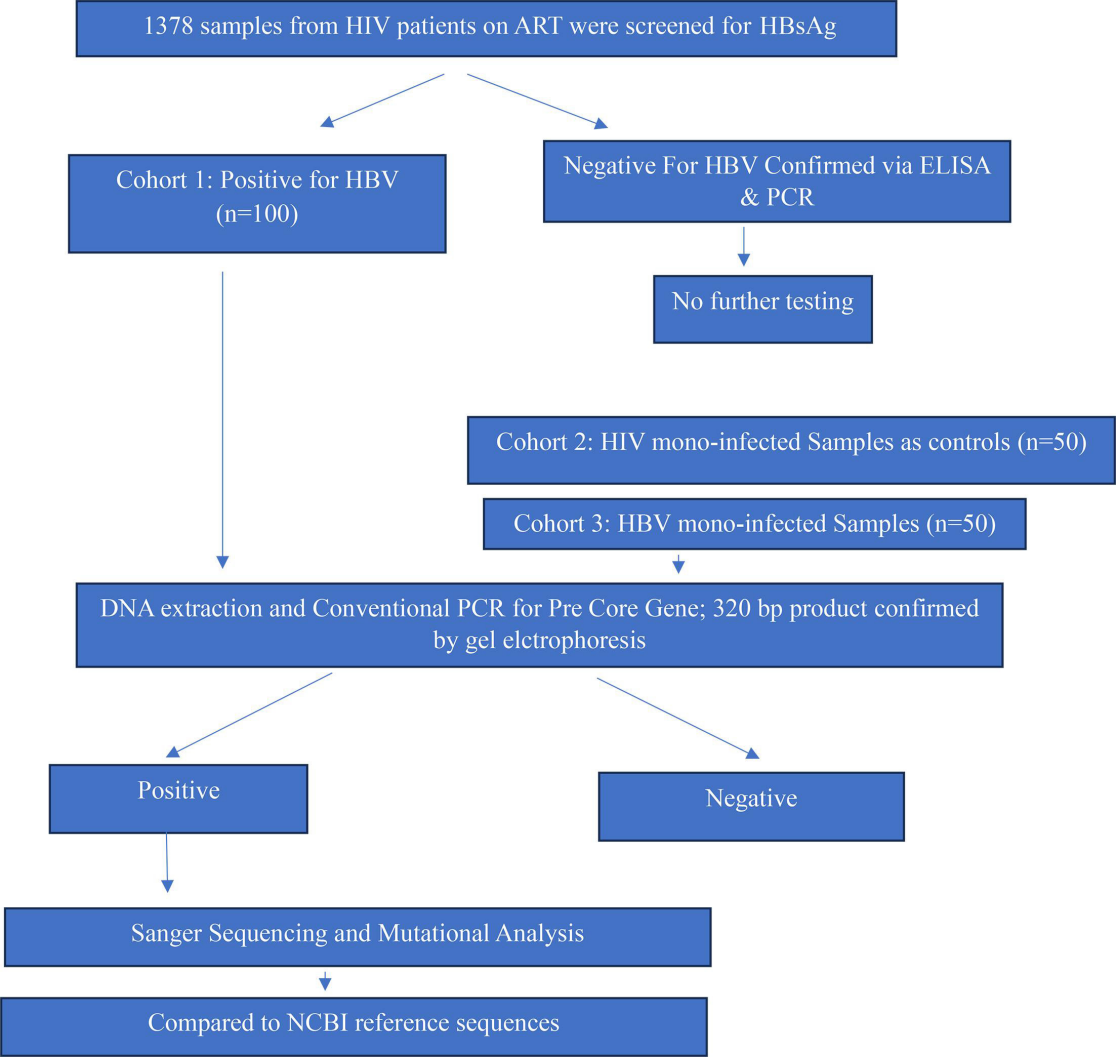
Flow chart showing selection and processing of HIV/HBV co-infected and HBV mono-infected patient samples for HBV PC region mutation analysis.

A cohort of 100 individuals co-infected with HBV-HIV (cohort 1), along with 50 HIV mono-infected (cohort 2) and 50 HBV mono-infected (cohort 3) patients (Fig. 1), was enlisted from various hospital settings, including outpatient departments, hospital wards, the Anti-Retroviral Treatment clinic, and the ICTC (Department of Microbiology) at J.N. Medical College and Hospital, A.M.U., Aligarh. HIV patients included both treatment-naïve individuals and those undergoing antiretroviral therapy.

The inclusion criteria included individuals aged between 18 and 65 years, the confirmation of the presence of HBsAg and the absence of autoimmune hepatitis, alcoholic hepatitis, drug-induced hepatitis or co-infections involving both HBV and hepatitis C virus (HCV), as well as other hepatitis viruses like hepatitis A virus (HAV) or hepatitis E virus (HEV). The study excluded patients with recent infections, surgeries or traumas within the preceding 2 months and those with renal insufficiency or other acute or chronic inflammatory conditions. Furthermore, the study did not include individuals who declined to provide consent.

### Collection of specimens

Venous blood samples (5–10 ml) were taken after the patients gave their informed written consent. Serum isolation was carried out through centrifugation, and the collected serum was stored at −20°C for subsequent analysis.

### Serological investigations

Patients presenting with symptoms associated with hepatitis, in the context of HIV, underwent screening for various hepatitis viruses, namely, HAV, HBV, HCV and HEV. Commercially available ELISA kits were used for screening, which included assays for HBsAg, anti-HCV, anti-HAV IgM and anti-HEV IgM. Individuals who tested positive exclusively for HBV were chosen to participate in the study. For those co-infected with both HIV and HBV, additional testing was conducted to ascertain the presence of HBe antigen. Every diagnostic process was carried out according to the instructions supplied by the kit manufacturers.

Patients were categorized as having mild, moderate and severe liver disease based on various factors, including the duration of illness, serum examination results and biochemical analysis as defined by Hiba *et al.* [20].

### HBV DNA extraction, PCR amplification and Sanger sequencing

HBV DNA was extracted from 200 µl of serum using the QIAamp DNA Blood Mini Kit (Qiagen, Germany), following the manufacturer’s protocol. Briefly, serum samples were first lysed by incubation with proteinase K and lysis buffer at 56 °C to ensure complete viral capsid disruption. After lysis, ethanol was added to facilitate DNA binding to the spin column membrane. The mixture was then loaded onto the QIAamp silica membrane column and centrifuged. Bound DNA was washed in two steps using provided buffers to remove contaminants. Finally, DNA was eluted in 200 µl of elution buffer and stored at –20 °C until further use for PCR and sequencing. The PCR for the PC region was carried out using Takara Master Mix (Takara, Shiga, Japan) with the following primers: forward 5′-TATTAGGAGGCTGTAGGCAT-3′ and reverse 5′-AGAATAGCTTGCCTGAGTGC-3′. Cycling conditions for amplification included an initial denaturation at 94 °C for 3 min, 39 cycles of denaturation at 94 °C for 30 s, annealing at 58 °C for 50 s and extension at 72 °C for 50 s, followed by a final extension at 72 °C for 5 min. The 320 bp amplicon was confirmed by 1.5% agarose gel electrophoresis. Gel electrophoresis settings were adapted from the protocol described by [20]. PCR products were submitted for Sanger sequencing (Eurofins Genomics Pvt. Ltd.). Resulting sequences were compared to HBV reference strains using NCBI’s Nucleotide blast tool.

### Viral strain characterization, infection quantification and data quality control

HBV genotyping was conducted based on sequence data from the PC/core region using the NCBI HBV genotyping tool, and findings were validated through phylogenetic analysis with mega X software. HBV viral loads were quantified using a commercial real-time PCR assay (Altona Diagnostics, Germany), with a detection limit of 6.31 IU ml^−1^. Each sample was tested in duplicate for accuracy. HIV status had been previously confirmed by ELISA and PCR, while HBV infection was verified at the time of recruitment through HBsAg screening using a commercial ELISA kit (QUALISA, India). To ensure data integrity, positive and negative controls were used throughout the PCR and sequencing workflows. All DNA extractions adhered to strict protocol compliance. Outlier laboratory values were flagged and re-evaluated either by repeat testing or independent clinical review before inclusion in the final dataset.

### Sequence alignment and phylogenetic analysis

Sequence alignment for all study sequences was performed using the UGENE software and the ClustalW method (Fig. S1, available in the online Supplementary Material). The wild HBV consensus sequence, identified from prior literature, was adopted as the reference sequence. Each sequence was then aligned with the reference sequence, as detailed above, for mutation analysis. The aligned sequences were imported into UGENE version 2.0, where the neighbour-joining method was employed to construct the phylogenetic tree. To assess the reliability of the tree’s branching patterns, a bootstrap analysis was performed. Reference sequences retrieved from GenBank, identified by accession numbers, were also included in the tree for comparison. The generated phylogenetic tree was visualized and annotated using iTOL. The isolates were colour-coded based on their geographic origin and genotype classification. The reference sequence was highlighted to serve as a baseline for evolutionary comparison.

### HBV genotyping and mutational analysis

For genotyping, a phylogenetic analysis was conducted using the entire pre-C/C region, which we amplified to a length of 320 bp, for all 27 HBV strains. The nt sequences of 27 HBV strains were compared with those of 19 reference strains, each representing genotypes A through H, including 23 genotype D strains retrieved from GenBank [accession numbers LC519791 (A), AB219428 (B), GQ924620 (C), NC_003977 (D), AB106564 (E), AY090458 (F), AB064313 (G), AY090454 (H), KP243417 (D), KF798282 (D), MK507913 (D), KM524355 (D), MK598650 (D), MT591278 (D), MF925407 (A), MN702657 (D), GU456671 (D) and GQ205384 (D)]. Mutations were identified through comparisons with the consensus sequence of HBV strains in our cohort and the 19 reference strains.

### Statistical analysis

Statistical analysis was performed using IBM SPSS Statistics version 19. The Shapiro–Wilk test was used to assess the normality of continuous variables. Normally distributed data were presented as means±sd and compared using independent-samples t-tests or one-way ANOVA. Non-normally distributed data were expressed as medians with interquartile ranges and analysed using the Mann–Whitney U test or Kruskal–Wallis test, as appropriate.

Associations between categorical variables – including HBsAg status and HIV transmission routes, HBeAg positivity, CD4 immunosuppression status and liver injury markers [ALT (alanine transaminase), AST (aspartate transaminase), APRI (AST to platelet ratio index) and FIB-4 (Fibrosis-4) indices] – were assessed using the chi-square test. One-way ANOVA was applied to compare means across liver disease severity groups (mild, moderate and severe).

A *P*-value of <0.05 was considered statistically significant. To identify predictors of severe liver disease, multivariable logistic regression was conducted, incorporating covariates such as age, sex, HBV genotype and HIV status. Sensitivity analyses were carried out to assess the robustness of key findings.

## Results

### Clinical and laboratory assessment

In the HBV-HIV co-infected group, which comprised 100 patients with varying degrees of HBV severity, nearly half (45%) belonged to the moderate category (Table 1). Males made up the majority of participants in the ‘mild’ (58.1%), ‘moderate’ (62.2%) and ‘severe’ (62.5%) cases, with the respective mean ages of 30±1, 36±1 and 41±1 years (range from 18 to 65 years). A statistically significant difference was found across the three groups (***P*<0.001**), suggesting that disease severity increases with age.

**Table 1.** Demographic, virological and laboratory-based parameters of HBV-HIV co-infected patients (*n*=100)

HIV co-infected	Category 1	Category 2	Category 3	*P*-value
	Mild (*n*=31)	Moderate (*n*=45)	Severe (*n*=24)	
Age (mean±sd)	30±1	36±1	41±1	*P*<0.001
Gender (M/F)	18/13	28/17	15/9	*P*=0.88
Laboratory evaluation				
ALP (IU l^−1^)	170.2	145.3	199.82	*P*=0.02
ALT (IU l^−1^)	59.3	51.20	84.54	*P*=0.01
AST (IU l^−1^)	41.5	47.59	87.80	*P*<0.001
Blood urea (mg dl^−1^)	15.75	12.86	20.92	*P*=0.03
Haemoglobin (g dl^−1^)	12.2	12.06	12.72	*P*=0.12
Platelets (per microlitre)	244,210	180,178	132,000	*P*<0.001
Creatinine (mg dl^−1^)	0.75	0.76	1.02	*P*=0.04
Bilirubin (mg dl^−1^)	0.96	0.79	1.32	*P*=0.02
TLC (per microlitre)	6,978	8,760	7,315	*P*=0.05
Special parameters				
APRI	0.30	0.55	1.05	*P*<0.001
CD4 (per microlitre)	347	385	321.53	*P*=0.19
FIB-4	0.71	1.42	3.14	*P*<0.001
HBeAg positive	3	3	4	*P*=0.72
HBV DNA (log copies per millilitre)	93,106,513.7	37,118,508.09	421,684,814.8	*P*=0.002

ALP, alkaline phosphatases; ALT, alanine transaminases; AST, aspartate transaminases; HBeAg, HBV-e antigen; TLC, total leucocyte count.

Table 1 shows the demographic, virological and laboratory-based parameters of HBV-HIV co-infected patients.

The mean APRI (*P*<0.001) and mean FIB-4 Index (***P*<0.001**) for liver fibrosis increased parallelly to the ascending categories, and their significant increase with severity suggests worsening liver fibrosis. An APRI score of >0.5 and a Fib-4 score of ≥1.45 were used to define liver fibrosis (Table 1).

Out of the 100 HBV-HIV co-infected patients, three (9.7%) had HBeAg-positive hepatitis in the mild case category, three (6.7%) in the intermediate severity group and four (16.7%) in the severe case category. HBV DNA (*P*=0.002) shows a significant difference, with the highest viral load in the severe group, supporting the role of HBV replication in disease progression.

### Amplification and sequencing of the HBV PC gene

**Fig. 2.**
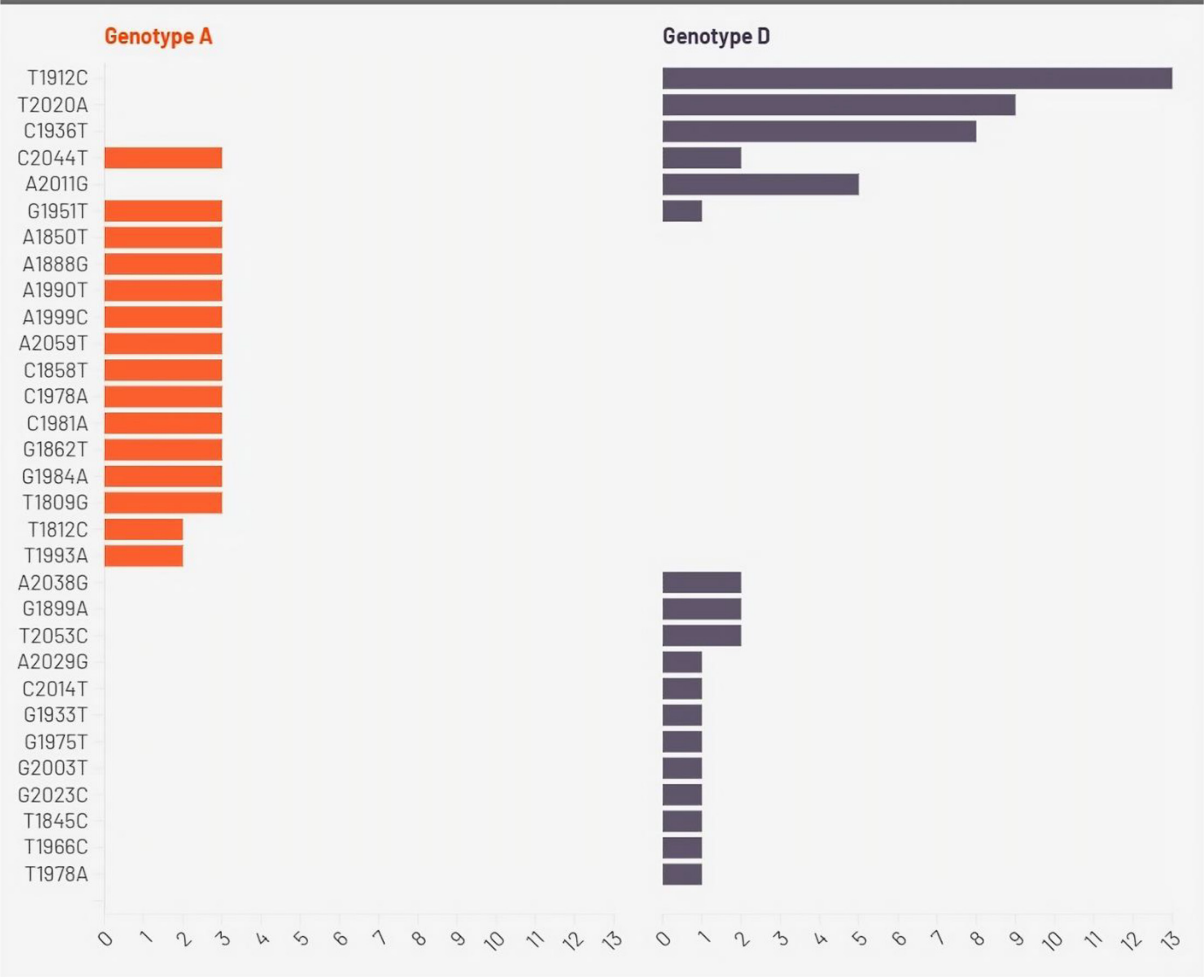
Heatmap showing the presence of mutations across different genotypes.

The PC region was successfully amplified and sequenced in a total of 27 samples, i.e. 16 HBV-HIV co-infected and 11 HBV mono-infected samples, as shown in Fig. S2. Moreover, a detailed examination of sequences from the PC region was conducted, comparing them with sequences sourced from the GenBank as reference sequences. Interestingly, our analysis revealed the presence of HBV genotypes D and A across all study samples, with genotype D comprising 24 (88.9%) and genotype A accounting for 3 (11.1%) of the total samples analysed. Fig. 2 shows the heatmap showing the presence of mutations across different genotypes in the mutations observed in the HBV PC gene for both genotype A (*n*=3) and genotype D (*n*=24) samples.

### Analysis of the sequences

All the HBV samples sequenced were of genotype D, except three of them which belonged to genotype A.

#### Genotype D

Among the 24 sequences analysed, the occurrence of mutations varied. Specifically, the T1847C mutation was observed in only one patient, while A1901G was identified in two patients. Moreover, T1912C was found in 13 patients, with G1935T, C1938T, A1953G, T1968C, G1977T, T1980A and T1995A each present in one patient. Similarly, G2005T, A2013G, A2031G, A2040G, C2046T and T2055C mutations exhibited varying frequencies, being present in one to nine patients. Interestingly, the novel mutation C2016T was exclusive to only one patient.

#### Genotype A

**Fig. 3.**
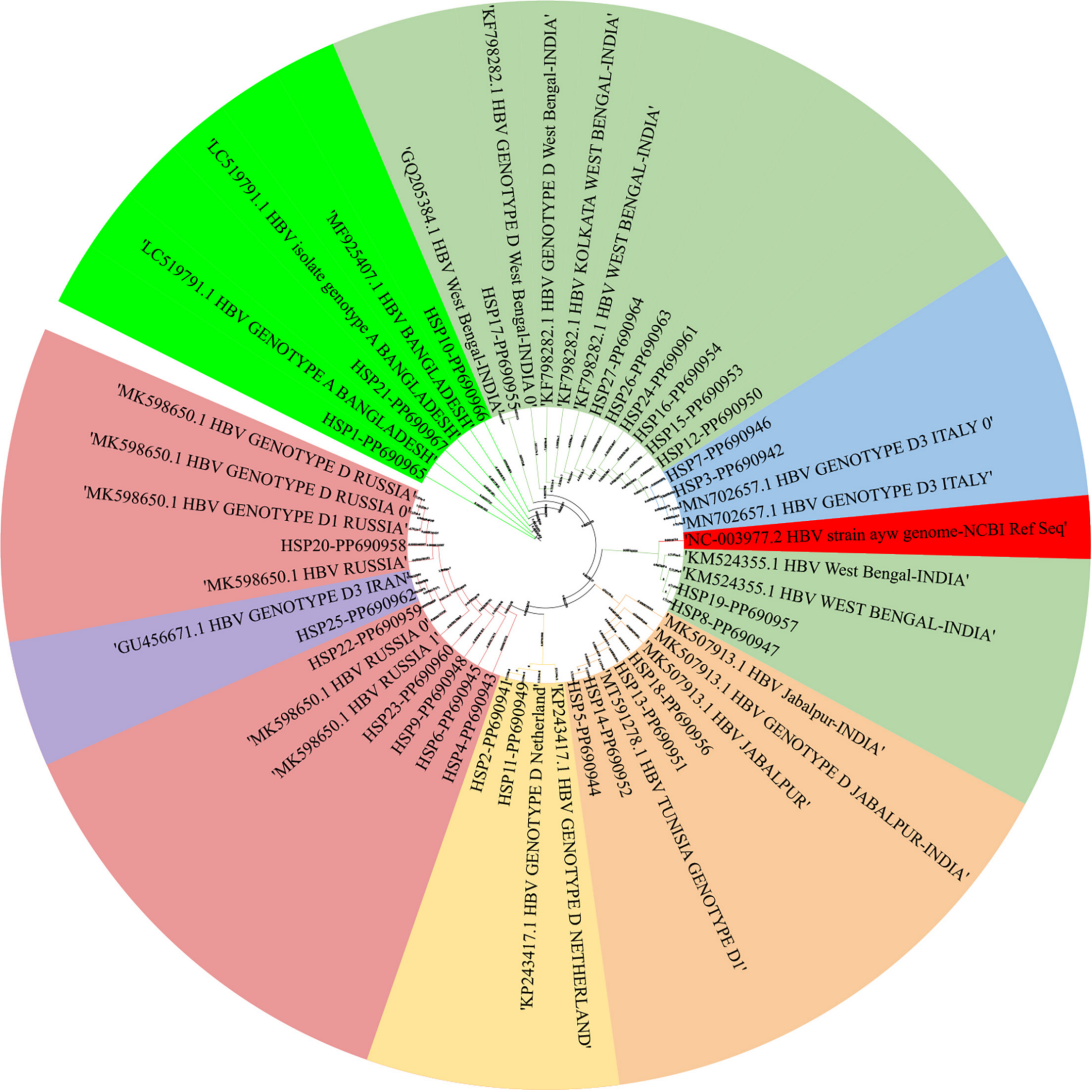
This phylogenetic tree represents the evolutionary relationships among HBV isolates from different geographic locations and genotypes. The tree was constructed using iTOL based on nt sequence alignments. Highlight: represents the reference sequence (NCBI RefSeq) used for comparison. Genotype A is highlighted in green, and all other highlighted colours represent genotype D samples from different geographical regions around the world. The bootstrap values (black circles at nodes) indicate the reliability of branching patterns.

The three genotype A sequences were compared to LC-519791 (Bangladesh), MF-925407 (Bangladesh) and LC-519791 (Bangladesh) as a reference strain. In these sequences, mutations including T1811G, A1852T, C1860T, T1864G, A1890G, A1953G, C1980A, C1983A, G1986A, A1992T, A2001C, C2046T and A2061T were identified in all three samples. Additionally, the T1814C mutation was present in two of the samples. Fig. S3 displays the phylogenetic tree derived from multiple sequence alignments along with reference sequences. Fig. 3 represents the phylogenetic tree constructed using iTOL (Interactive Tree of Life) based on nt sequence alignments.

**Fig. 4.**
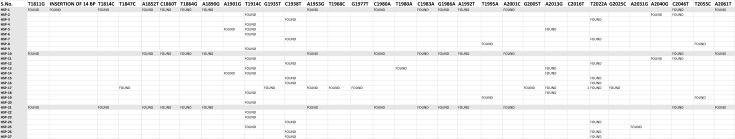
Summary of observed mutations in the PC region based on sequence alignments (*n*=27). This figure illustrates the pattern and frequency of key nt substitutions, including G1896A and A1762T/G1764A, across the patient cohort.

Mutations were detected at various positions, with different frequencies observed between the two genotypes. For genotype A, mutations were predominantly present, with 100% frequency observed for most mutations. In contrast, genotype D exhibited a more varied pattern, with mutations detected at lower frequencies. Notably, certain mutations, such as T1912C (13; 48.1%), T2020A (9; 33.3%) and C1936T (8; 29.6%), were predominant and solely found in 54.1%, 37.5% and 33.3% of genotype D samples, respectively. The C2044T mutation (5; 18.5%) was found in genotype A (3; 100%), as well as D (2; 8.3%), while others, like C1858T and A1888G, were prevalent in genotype A (100%). The assessment of distribution of HBV PC mutations among the genotypes (Fig. 2) demonstrated the presence of a statistically significant difference in the occurrence of T1809G, T1812C, A1850T, C1858T, G1862T, A1888G, G1951T, C1978A, C1981A, G1984A, A1990T, T1993A, A1999C, C2044T and A2059T mutations amongst individuals infected with the HBV genotypes A and D (*P*<0.05, Fisher’s exact test). All HBV/A strains (3 out of 3, 100%) had T1809G, A1850T, C1858T, G1862T, A1888G, G1951T, C1978A, C1981A, G1984A, A1990T, A1999C, C2044T and A2059T mutations (Fig. 2). Sequence alignment of the PC region revealed specific mutations among the 27 co-infected individuals. These findings are illustrated in Fig. 4, which summarizes mutation profiles

## Discussion

Mutations in the PC region can affect different aspects of HBV infection, like HBeAg status, viral replication, nucleocapsid formation and packaging of pgRNA [21,25]. Though there are differences in infecting genotypes and patient factors like HIV co-infection, ethnicity and location, various types of PC mutations affecting disease progression in chronic patients have been identified [26]. According to certain research, individuals infected with HBV who have PC mutations have a much-increased risk of developing HCC and severe liver disease [27]. In this study, we selected the PC region to assess mutations in HBV DNA sequences. We identified 30 nt variations in the PC region from HBV sequences of HIV/HBV co-infected patients.

HBV infection remains a significant health concern in North India, where the prevalence of HBV is notably high [28]. Different PC mutations impacting the course of the disease in chronic patients have been identified, despite variations in the infecting genotypes and patient variables such as location, ethnicity and HIV co-infection [26]. The most common genotype in the current investigation was HBV genotype D (88.9%), which is consistent with other findings from past research conducted in India [29,32]. According to the majority of studies published from India, 30–40% of HBV isolates are genotype A, and 50–60% are genotype D [13,29]. However, only 11.1% of the samples in this study were assigned to genotype A.

Sanger sequencing of 27 HBV samples showed a high prevalence (48.1%) of T1912C PC mutations; these were displayed in 54.1% of genotype D strains. This is consistent with findings from Sharma *et al*. [33], who also observed a high frequency of PC mutations among Indian patients predominantly infected with genotype D HBV. These mutations have been associated with altered HBeAg expression and may contribute to the clinical variability observed in chronic HBV infections [34].

In our cohort, 90% of HBV isolates were HBeAg-negative, a finding that differs from several earlier studies reporting higher HBeAg positivity, especially in treatment-naïve patients. This high prevalence of HBeAg negativity might reflect the chronic phase of infection, immune-mediated clearance or selective pressure in co-infected individuals. The T1912C mutation, commonly observed in genotype D cases in our study, could contribute to reduced HBeAg expression. However, due to the small sample size and lack of temporal data on infection status, these findings should be interpreted with caution. Previous studies have linked such mutations to the clinical severity of HCC [9]. All three of our genotype A samples (100%) tested positive for T1809G HBV PC mutations, and two of them (66.6%) also displayed T1812C. Distribution of these translational mutants was also discovered in an Ethiopian study population, including their HIV/HBV group (T1809G: 43.8%; T1812C: 45.8%), according to a study analysis by Belyhun *et al.* [35]. Contrary to our findings, however, the majority of these mutations were found in genotype D infections (T1809G: 78.6%; T1812C: 85.7%), and sparingly in genotype A samples (T1809G: 2.3%; T1812C: 3%).

The current investigation also showed that all of our genotype A patients had 100% rates of A1850T and C1858T HBV PC gene mutations; however, it should be highlighted that only three genotype A samples were identified and sequenced, making these results not statistically significant. PC mutations with substitutions C to T at position 1858 (corresponding to codon 15 without replacement of amino acid Pro) and A to T at location 1850 (Thr to Ser in codon 13) were found in genotype A. Similar findings were noted in a Spanish investigation, which additionally indicated that position 1858 is matched in the encapsidation signal; in certain variants, the presence of a C to T substitution in position 1858 resulted in the correct base pairing [36]. Also, in sync with our investigation, Mbamalu *et al.* discovered that the A1850T nt variation was associated with amino acid change/effect T to S (threonine to serine) [14]. This missense mutation contributed to 35.7% of their HBV A genotypes.

In contrast to our findings, earlier research indicated that C1858 was a highly selective variant for genotype C and was relatively common; the highest prevalence of C1858 was found in cases of HCC [37,40]. Comparing these results to those of other studies involving patients from Sweden and Vietnam revealed that our study did not yield results similar to those of other studies [37,38]. It would be worthwhile to conduct a large-scale study in order to compare the genotype characteristics across Southeast and East Asian countries.

The C1858T mutation found in our study emphasizes its role in HBV-related liver diseases, particularly HCC. This mutation, specific to HBV genotype A, could serve as a valuable biomarker for identifying individuals at higher risk of developing HCC. Understanding genotype-specific mutations like C1858T is crucial for better managing HBV infections and associated liver complications [14].

Likewise, the detection of the G1899A mutation (7.4%) in HBV also signifies an increased risk of HCC [14]. In our research, we were able to identify some mutations associated with HCC survival in the PC region, including G1899A, which may cause changes to the residues of amino acids. While G1899A has been extensively studied and is known to prevent the production of HBeAg, other mutations in the PC region have also been associated with HCC outcome, irrespective of their impact on codon mutation [41,42]. Further validation in diverse populations and laboratory-based functional studies is warranted to elucidate the mechanisms through which these mutations influence HCC progression and the distinction between mutation types in introducing codon mutations [12,43].

The G1899A mutation found in genotype D (2 out of 24; 8.3%) may also predict liver disease progression in CHB cases [44]. This suggests the importance of genotype-specific mutations in determining disease severity and guiding treatment decisions for affected individuals. Further research is needed to confirm this association and understand its implications for patient management.

In a study conducted on an Eastern Indian population, Chandra *et al.* reported that G1862T was found in 15 (83%) genotype A and 3 (17%) genotype D samples [45]. The mean viral load was lower in individuals with the G1862T mutation, and this mutation was more common in HBeAg-negative (21%) than in HBeAg-positive (9%) patients. Regardless of HBeAg status, the G1862T mutation was only discovered in HBV/A isolates (3; 100%) in our population and not in genotype D. Furthermore, there was no correlation found between this mutation and viral load. These results suggest that the G1862T mutation most likely contributes to the naturally occurring variability of HBV genotype A.

In this study, we found that only 10% of patients tested positive for the HBeAg. This aligns with previous research showing that the G1862T mutation reduces the expression of HBcAg and pre-C/C/HBeAg. This mutation leads to a decrease in HBeAg secretion, potentially triggering an immune response against hepatocytes and contributing to liver damage, as seen in our patients [46]. The G1862T mutation, common in sub-genotype A1, may heighten the risk of HCC [47]. Additionally, a further reduction in HBcAg expression is observed when G1862T and G1888A mutations coexist [48], indicating that while HBeAg secretion is not entirely abolished, it is significantly reduced by the presence of G1862T [44,49]. This highlights the importance of the G1862T mutation in altering viral protein expression and its potential impact on disease progression, including HCC.

T1845C, A1888G, G1933T, T1966C, G1975T, C1978A, T1978A, C1981A, G1984A, A1990T, T1993A, A1999C, G2003T, C2014T, G2023C, A2029G, A2038G, T2053C and A2059T are some of the additional HBV PC region mutations that were found. We searched the NCBI database globally and found no information about the mutations C1936T, A2011G, T2020A and C2044T. The only way to assess the clinical outcomes for the newly recognized mutations is to conduct additional research using a larger sample size.

These findings may hold clinical relevance for the management of HIV/HBV co-infected individuals. Identification of specific PC mutations, particularly those associated with HBeAg negativity or elevated ALT/AST levels, could serve as molecular indicators of progressive liver damage.

## Conclusion

This study provides insight into the spectrum of HBV PC mutations and their potential associations with HIV co-infection and liver disease severity. The findings suggest that specific mutations within the PC region may be more prevalent in certain HBV genotypes and could be linked with elevated liver enzyme levels and clinical indicators of liver injury. Limitations include the relatively small sample size and the absence of longitudinal follow-up to assess the persistence or clinical progression associated with the detected mutations.

## Supplementary material

10.1099/acmi.0.000927.v4 Uncited Supplementary Material 1.

## Abbreviations

ALT: alanine transaminase
APRI: AST to platelet ratio index
AST: aspartate transaminase
BCP: basal core promoter
CHB: chronic hepatitis B
CLD: chronic liver disease
FIB-4: Fibrosis-4
HAV: hepatitis A virus
HBeAg: HBV-e antigen
HBsAg: HBV surface antigen
HBV: hepatitis B virus
HCC: hepatocellular carcinoma
HEV: hepatitis E virus
HIV: human immunodeficiency virus
iTOL: Interactive Tree of Life
PC: pre-core
