# Supplementary material for: Molecular analysis of HBV pre-core gene mutations in patients co-infected with HIV at a tertiary care hospital in North India
Source: Access Microbiol. 2025 Aug 5;7(8):000927.v4. doi: 10.1099/acmi.0.000927.v4 (PMC12322836; doi:10.1099/acmi.0.000927.v4)
Supplement: Uncited Supplementary Material 1. [file acmi-7-00927-s001.pdf]

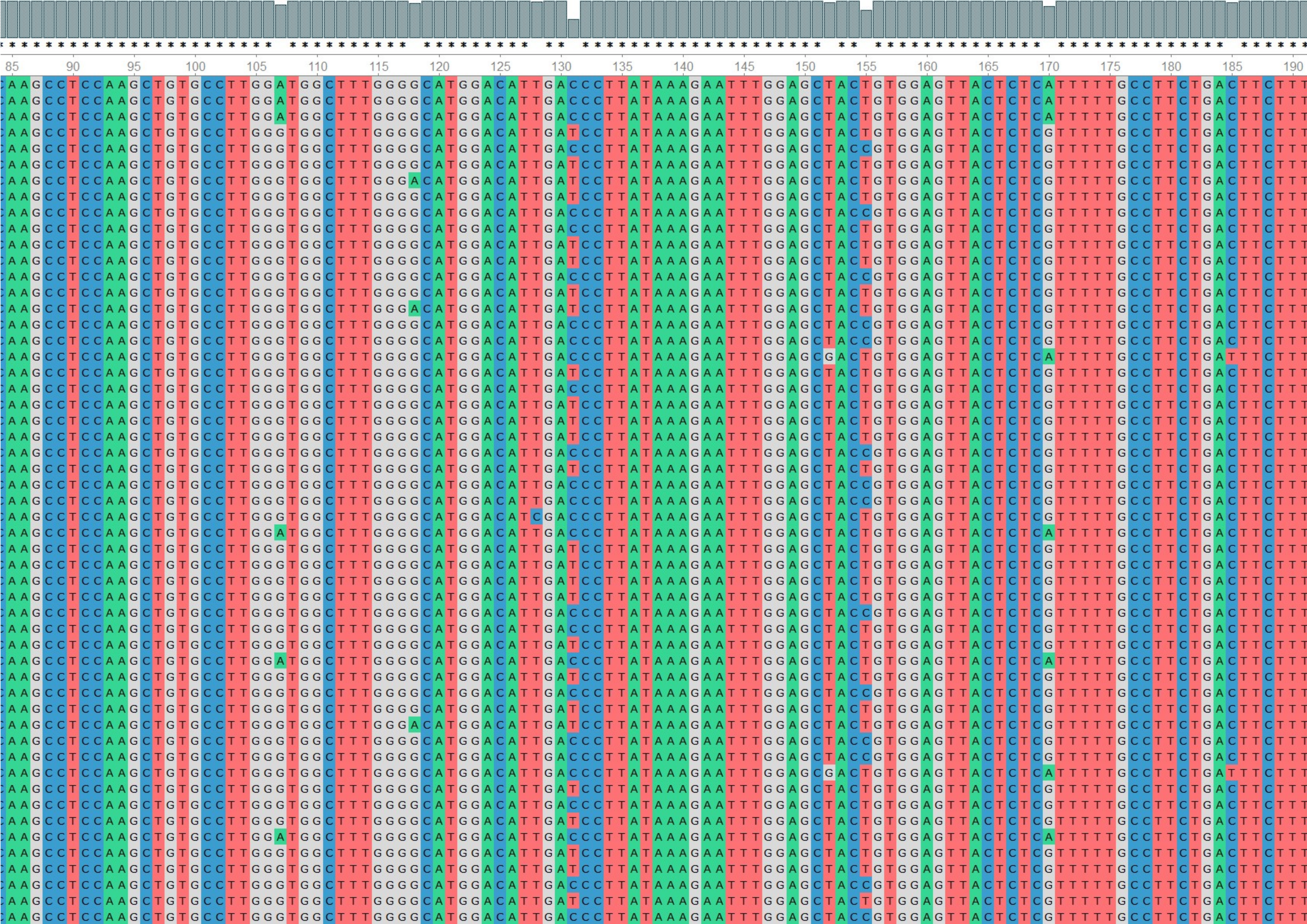
85 90 95 100 105 110 115 120 125 130 135 140 145 150 155 160 165 170 175 180 185 190

Supplementary Figure 1: Multiple Sequence Alignment of study sequences was performed using the UGENE software and the clustal W method. This alignment provides insights into genotypic variations, evolutionary relationships, and potential mutation hotspots within the HBV genome.

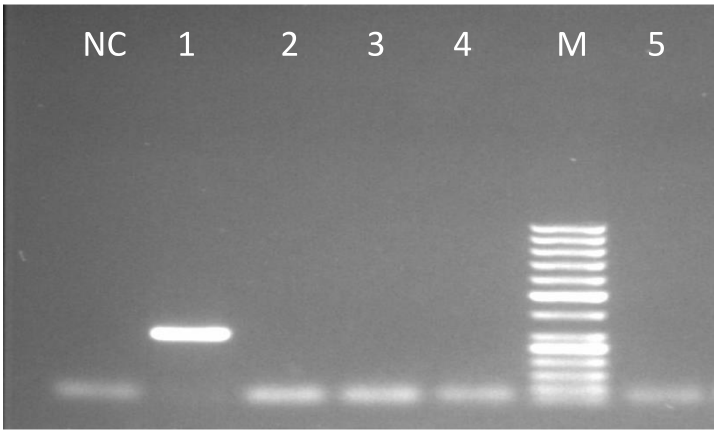

Supplementary Figure 2: Agarose gel electrophoresis of HBV Pre Core Gene;PCR product of 321 bp. Lane M:50 bp DNA ladder; lane NC: Negative Control; Lane 1: positive sample; Lane 2,3,4,5: Negative samples

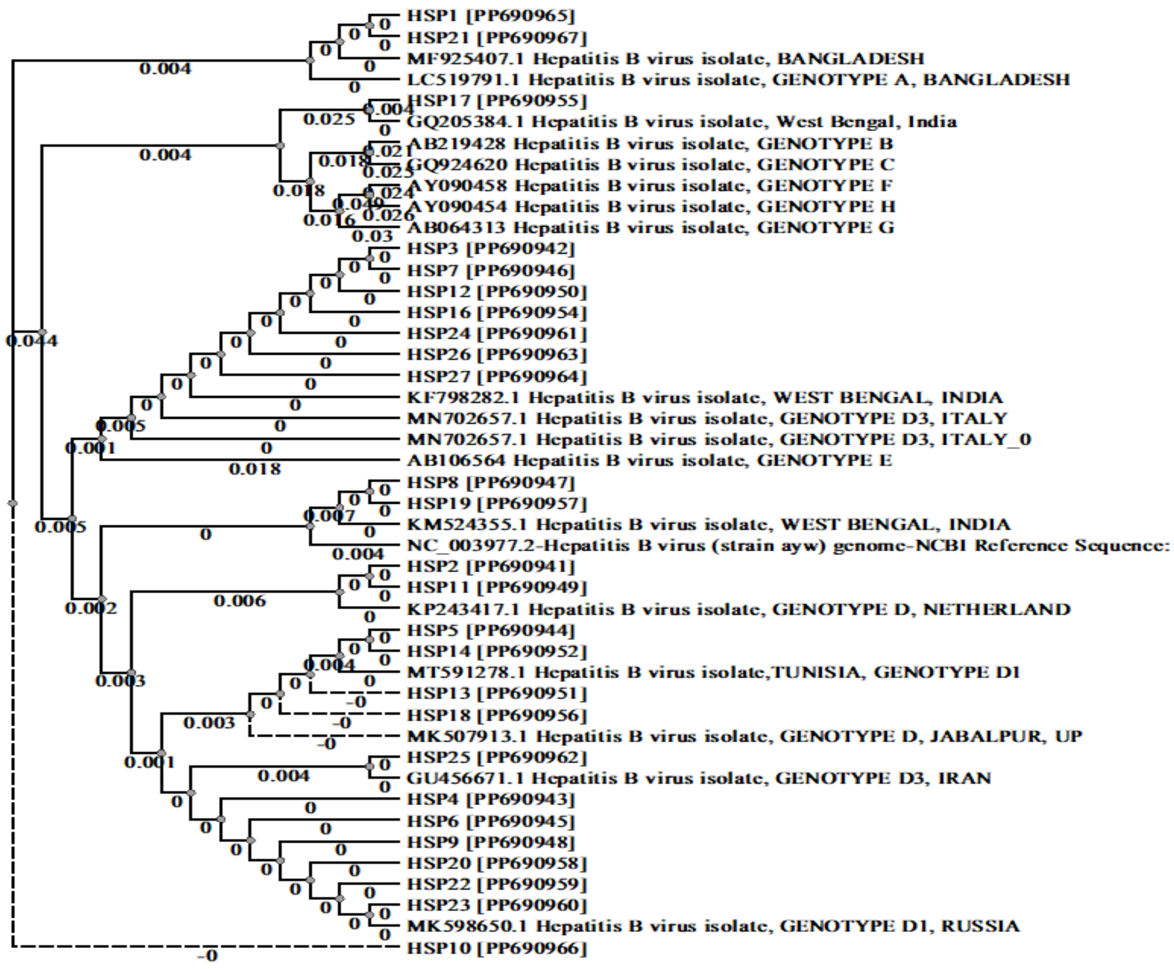

Supplementary Figure 3. For genotyping, a phylogenetic analysis was conducted using the entire pre-C/C region, which we amplified to a length of 320 base pairs for all 27 HBV strains. The nucleotide sequences of these 27 HBV strains were compared with those of 19 reference strains, each representing genotypes A through H, including 23 genotype D strains retrieved from the GenBank [Accession numbers LC519791 (A), AB219428 (B), GQ924620 (C), NC_003977 (D), AB106564 (E), AY090458 (F), AB064313 (G), AY090454 (H), KP243417 (D), KF798282 (D), MK507913 (D), KM524355 (D), MK598650 (D), MT591278 (D), MF925407 (A), MN702657 (D), GU456671 (D), and GQ205384 (D)]. Mutations were identified through comparisons with the consensus sequence of HBV strains in our cohort and the 19 reference strains. Phylogenetic trees were constructed using the neighbour-joining method with UGENE version 2.0.
